# FEV_6_ as screening tool in spirometric diagnosis of obstructive airway disease

**DOI:** 10.4103/0970-2113.63608

**Published:** 2010

**Authors:** P. Adithya Malolan, Vishak Acharya, B. Unnikrishnan

**Affiliations:** *Kasturba Medical College Hospital, Mangalore, India*; 1 *Department of Chest Diseases, Kasturba Medical College Hospital, Mangalore, India*; 2 *Department of Community Medicine, Kasturba Medical College Hospital, Mangalore, India*

**Keywords:** Forced vital capacity, forced expiratory maneuver to six-second duration, obstructive airway disease, spirometry

## Abstract

**Context:**

The use of spirometry is currently limited to the diagnosis of obstructive airway disease for tertiary centers mainly because of the unmet need for technical expertise and funding. Use in primary care asks for a simpler and cost-effective screening tool for obstructive airway disease.

**Aim:**

To estimate the efficacy of FEV_6_ against the current standard of FVC in the spirometric diagnosis of obstructive airway disease.

**Setting and Design:**

The Pulmonary Function Laboratory of a tertiary care hospital in Coastal South India. It was a descriptive study.

**Materials and Methods:**

We analyzed 150 serial patients on ATS standardized spirometers. The patients were classified into normal subjects and those with airway obstruction, further categorized as mild, moderate and severe and those with mixed defect. Those with obstruction were also classified as having reversible and irreversible defects.

**Statistical Analysis:**

Data was analyzed using SPSS Software (v.11.5), statistical test ANOVA and Pearson correlation was done and P less than 0.05 considered statistically significant.

**Results:**

FVC and FEV_6_ showed a linear correlation in all subjects. The difference in means was statistically significant in all subjects. The sensitivity and specificity of FEV_1_/FEV_6_ in comparison to FEV_1_/FVC were both found to be 100%.

**Conclusion:**

FEV_6_ is an excellent screening tool in the diagnosis of airway obstruction but, there is a necessity for further research to confirm our findings. There is also a need for reference values in an Indian setting to find out the efficiency of this new parameter. Our sample size is relatively small and comprises of a very high proportion (70%) of subjects with airway obstruction and so our results may not be applicable for use in general population.

## INTRODUCTION

The acceptability criteria for forced vital capacity (FVC) maneuver during PFT have been previously described by American Thoracic Society (ATS): Duration of exhalation should be at least six seconds during which a minimum one second plateau could be reached.[1]

The FVC also has the problem of being dependent on expiratory time in individuals with airway obstruction and in older persons.[2] Studies have suggested that reducing the forced expiratory maneuver to six-second duration (FEV_6_ ) could replace the FVC maneuver in the diagnosis of airway obstruction.[3,5]

Our aim was to assess the correlation of FVC with FEV_6_ and the sensitivity and specificity of FEV_1_/FEV_6_ as compared to that of FEV_1_/FVC in the evaluation of airway obstruction.

## MATERIALS AND METHODS

The study, carried out in the Pulmonary Function Laboratory of a tertiary care hospital in south India, began after approval of institutional ethics committee and informed consent from all the subjects. Data from 150 serial patients referred to the lab from the Chest clinic of the same hospital was analyzed. Spirometry was carried out on a Collins Eagle Flow based spirometer. The system was incorporated with Collins Plus-SQL2000 software for spirometry. The software had the provision to calculate FEV_6_ in addition to FVC in the same manoeuvre. Patients were asked to withhold any rescue medication (short-acting bronchodilators, long-acting bronchodilators or caffeine derivatives) 12 hours prior to the spirometry. Subjects were tested while seated and all the procedures were carried out in accordance with ATS criteria.[6] Height was measured to the nearest centimeter and weight was rounded off to the nearest kilogram.

Patients were categorized as having "airway obstruction" or "no airway obstruction" by using FEV_1_/FVC as the gold standard for diagnosis. Those with airway obstruction were further categorized as having a purely obstructive defect or mixed defect (obstruction with restriction). Those with spirometry showing pure restriction were excluded from the study as the correlation between FVC and FEV_6_ in those with restriction is yet to be validated and beyond the scope of our study. The severity of airway obstruction was graded into three categories: Mild (FEV_1_ 80-60% predicted), Moderate (FEV_1_ 60-40% predicted), Severe (FEV_1_ less than 40% predicted).[7] Patients were also categorized as having reversible or irreversible airway obstruction with reversibility defined as a 12% and second 200 ml improvement after administration of a short acting bronchodilator (Salbutamol 100 microgram via Metered Dose Inhaler).

The data, thus collected, was fed into Microsoft Excel. It was analysed using Statistical Package for Social Sciences 11.5 for Windows. Pearson correlation and ANOVA were used for statistical analysis. Data were reported as means plus/minus standard deviation.

## RESULTS

Baseline characteristics of the study population are given in Table 1. The sex distribution of the study subjects were 51.3% males. In all, 74.03% of the males and 67.12% of females presented with abnormal spirograms, either with obstruction or a mixed defect.

**Table 1:** Baseline characteristics

Characteristic	Mean ± SD
Age (yrs)	44.59 ± 18.18
Weight (kg)	59.21 ± 11.79
Height (cm)	160.49 ± 10.24
BMI (kg/m^2^)	23.02 ± 4.26
FEV_1_ (l)	2.07 ± 1.03
FEV_6_ (l)	2.57 ± 1.15
FVC (l)	2.58 ± 1.15
FEV_1_/FEV_6_%	79.86 ± 13.99
FEV_1_/FVC %	79.47 + 14.19
FVC-FEV_6_(ml)	13.20 ± 19.71

The difference between the FVC and the FEV_6_ values was found to be 13.20 plus/minus 19.71 ml with a range of 0-150 ml. Only in a solitary case, the difference was greater than 100 ml. The distribution of all subjects according to FVC-FEV_6_ is given in Table 2.

**Table 2:** Distribution of all subjects according to FVC-FEV_6_(n = 150)

FVC-FEV_6_(ml)	N	%
0	45	30
1-20	87	58
21-40	9	6
41-60	4	2.67
61-80	2	1.33
81-100	2	1.33
>100	1	0.67
Total	150	100

When those with no airway obstruction were considered separately, it was found that the difference between FVC and FEV_6_ was 7.73 plus/minus 7.43 ml with a range of 0-40 ml. However, when those with airway obstruction were considered, the difference increased to 15.47 plus/minus 22.60 ml with a wider range of 0-150 ml. This difference in means between those with and without airway obstruction was found to be statistically significant. Among those with airway obstruction, the difference was found to increase with increasing severity Table 3. When ANOVA was carried out with FVC and FEV_6_, the difference in means was found to be significant in all the subjects irrespective of the obstruction.

FVC and FEV_6_ showed a linear correlation in all the subjects including those with reversible and irreversible obstruction and those with a mixed defect [Table 4].

**Table 3:** Variation of FVC-FEV_6_ according to severity

Presentation	n	FVC-FEV_6_ (ml)		
		Mean ± SD	Max.	Min.
Normal	44	7.73 ± 7.43	40	0
Mild obstruction	47	13.62 ± 14.81	70	0
Moderate obstruction	18	18.33 ± 21.49	90	0
Severe obstruction	19	23.68 ± 39.75	150	0
Mixed defect	22	10.00 ± 15.43	60	0

**Table 4:** Correlation between FVC and FEV_6_

	Correlations	FEV_6_	FVC		
FEV_6_	Pearson correlation	1	1.000(**)
	Sig. (2-tailed)		0.000
	N	150	150
FVC	Pearson correlation	1.000(**)	1
	Sig. (2-tailed)	0.000	
	N	150	150

** Correlation is significant at the 0.01 level (2-tailed)

Upon comparison of the unadjusted values of FEV_1_/FEV_6_ to FEV_1_/FVC for diagnosing airway obstruction, both sensitivity and specificity of FEV_1_/FEV_6_ were 100%. The positive predictive value as well as negative predictive value were 100% as well.

## DISCUSSION

Guidelines state that airway obstruction is defined by a low FEV_1_/FVC. Therefore, a fault in the measurement of either of these values will lead to misclassification. To avoid errors in measurement of the FVC, the 1994 ATS recommendation stated that, to be considered acceptable, each maneuver should last until a plateau is achieved on the volume–time graph.[6] Patients with airway obstruction often fail to meet the end of test (EOT) criterion defined by a less than-20 ml change in the final two seconds of the maneuver.[8]

Failure to attain acceptable EOT plateaus is relatively common in clinical practice. This may be due to a variety of reasons like, lack of proper technical training, poor motivation of subjects to keep blowing, a faulty spirometer, subjects with severe disease etc. These in turn lead to an under-estimation of the FVC value leading to misclassification. The closer the FEV_1_/FVC value to Lower limit of normal, greater is the likelihood of missing early obstructive disease. We also found that, the difference in FVC and FEV_6_ increased with increasing severity. This factor could prove to be a drawback when it is attempted to replace FVC with FEV_6_ as it can lead to further misclassification.

With an accuracy of 100%, FEV_1_/FEV_6_ is an excellent alternative to FEV_1_/FVC in the diagnosis of airway obstruction. Comparing our results with those of Swanney *et al*. we obtained higher levels of sensitivity and specificity (100% and 100% in ours, vs. 95.0% and 97.4% in theirs).[2] This was also the case when compared to Vandevoorde *et al*. who obtained a sensitivity of 94% and specificity of 93.1%.[4] This may probably be because we used absolute values of FEV_1_/FEV_6_ in the diagnosis of obstruction without replacing them with reference values in the diagnosis of obstruction unlike the aforementioned studies.

The ATS recommends caution in the diagnosis of cases with values closer to the LLN because both results and estimation of thresholds can shift over to the other side very easily.[9] The interpretation of such results should include clinical information also. This difficulty in making the diagnosis was not evident in our study. This was probably due to the small sample size when compared to other studies[4,5,10] and the high prevalence of airway obstruction among our subjects. Further, many of our subjects were already diagnosed of airway obstruction and were on treatment and there were very few naïve patients.

Both FVC and FEV_6_ showed an excellent correlation further compounding the hypothesis that FEV_6_ is an excellent substitute in the diagnosis of airway obstruction. The disparities that could have arisen due to results being closer to normal limits can be avoided by the use of appropriate reference values for FEV_1_/FEV_6_. These have already been suggested by Hankinson *et al*.[11] for an American population and by Garcia-Rio F *et al*. for a European population.[12] However, reference values are currently unavailable for an Indian population and could limit the usage of FEV_6_ in clinical practice.

FEV_1_/FEV_6_ would be a very effective tool in the primary care scenario for screening and early detection of COPD among high risk patients, i.e, smokers over 45 yrs of age. The use of FEV_6_ instead of FVC has many distinct advantages: (a) it is easier for the technician and the patient, especially older patients with severe obstructive disease;[13] (b) there is a more discreet and precise end- of- test definition;[13] (c) there is some evidence that FEV_6_ is more reproducible than FVC;[2] (d) shorter maneuvers reduce the risk of syncope;[13] (e) it reduces the overall time taken to perform a spirometry;[13] (f) it reduces the need for spirometers with very accurate flow detection sensors as required at the end of the FVC maneuver.[2,13] All these advantages can make spirometry a relatively cost-effective and both patient/technician-friendly test to perform.

Most of the spirometers in manufacture, until now, are capable of measuring only FVC and not FEV_6_. This is a major handicap and probably a reason why such studies haven't been carried out in an Indian setting before.

Time was the major constraint we faced in this study as it had to be completed within a stipulated period of two months. This led to a limitation both in the number of subjects and the gamut of cases we might have obtained. We feel there were not enough cases near the LLN to compare sensitivity and specificity of FEV_6_ with respect to FVC.
